# Supplementary material for: The Insights into Mitochondrial Genomes of Sunflowers
Source: Plants (Basel). 2021 Aug 26;10(9):1774. doi: 10.3390/plants10091774 (PMC8466785; doi:10.3390/plants10091774)
Supplement: Supplementary file 1 [file plants-10-01774-s001.zip › plants-1317193-supplementary.pdf]

Table S1. Species specific SNP identified in H. occidentalis and H. tuberosus mitogenomes

Position in H. occidentalis mitogenome	H. occidentalis	H. tuberosus	H. grosseserratus	H. strumosus
48227	**T**	G	G	G
110132	**T**	G	G	G
145262	**C**	A	A	A
150145	**C**	G	G	G
150364	**C**	G	G	G
150571	**T**	G	G	G
173171	**C**	A	A	A
181893	**T**	G	G	G
199009	**C**	A	A	A
231470	**T**	G	G	G
240885	**C**	A	A	A
266122	**C**	A	A	A
**Position in H. tuberosus mitogenome**	**H tuberosus**	**H. occidentalis**	**H. grosseserratus**	**H. strumosus**
73577	**T**	G	G	G
92760	**G**	T	T	T
147829	**G**	T	T	T
153674	**C**	A	A	A
231384	**T**	G	G	G
257468	**T**	G	G	G

Table S2. SSR motif length distribution table. "x" – lower than threshold, "-"– not found.

H. occidentalis / H. tuberosus / H. strumosus / H. grosseserratus	Length of SSR							Total
Motifs	<5	5-7	8-10	11-15	16-30	31-40	>40	
T	x	x	1/1/1/0	1/1/2/1	-	1/0/0/1	0/1/0/1	3/3/3/3
A	x	x	1/1/2/1	0/2/1/1	-	-	0/0/0/1	1/3/3/3
G	x	x	2/1/1/2	0/0/1/0	-	-	-	2/1/2/2
C	x	x	0/0/0/1	-	-	-	-	0/0/0/1
TC	x	1/1/0/0	1/0/0/0	-	-	-	-	2/1/0/0
TA	x	2/2/2/3	-	-	-	-	-	2/2/2/3
AG	x	1/2/0/0	0/1/1/0	-	-	-	-	1/3/1/0
AT	x	1/1/1/1	-	-	-	-	-	1/1/1/1
CT	x	1/0/2/2	-	-	-	-	-	1/0/2/2
GA	x	0/0/1/1	-	-	-	-	-	0/0/1/1
ATT	x	1/1/0/0	-	-	-	-	-	1/1/0/0
GGTT	1/1/1/0	-	-	-	-	-	-	1/1/1/0
GAAA	1/1/0/1	-	-	-	-	-	-	1/1/0/1
TTAC	1/0/0/0	-	-	-	-	-	-	1/0/0/0
CTTA	0/1/0/0	-	-	-	-	-	-	0/1/0/0
GTAA	0/0/2/0	-	-	-	-	-	-	0/0/2/0
TTTC	0/0/1/0	-	-	-	-	-	-	0/0/1/0
AAAT	0/0/0/1	-	-	-	-	-	-	0/0/0/1
AACC	0/0/0/1	-	-	-	-	-	-	0/0/0/1
AAAGA	1/1/1/0	-	-	-	-	-	-	1/1/1/0

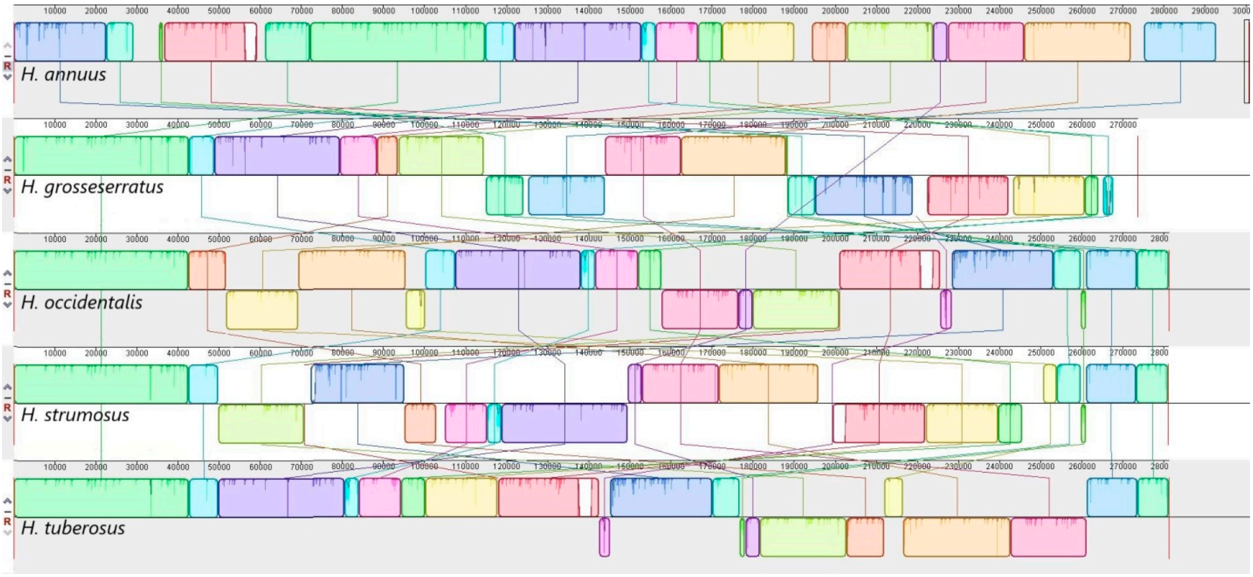

Figure S1. The progressive MAUVE alignment of complete mitogenomes between annual (*H. annuus*) and perennial (H. grosesserratus, *H. occidentalis*, *H. strumosus, H. tuberosus*) sunflower species

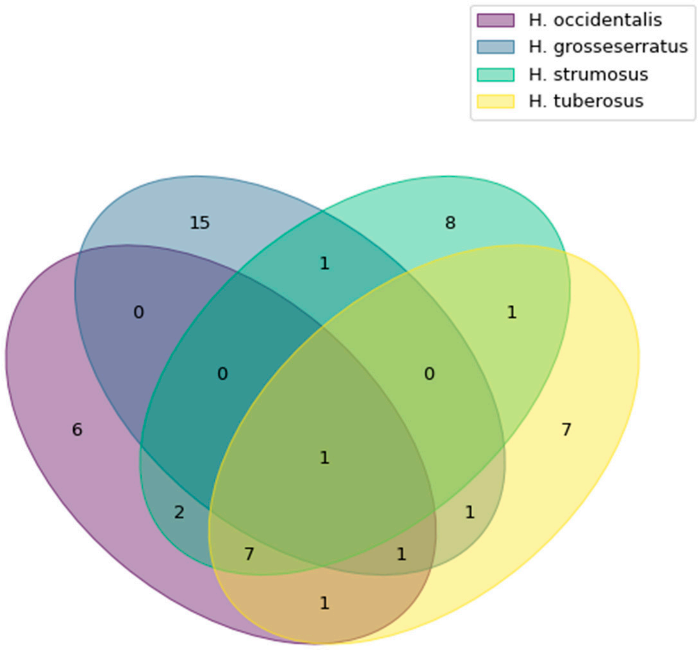

Figure S2. Venn diagram of SSRs comparison between species.
